# P-1004. Vancomycin Susceptibility Determination for Clostridioides difficile Varies by Brucella Agar Brand and Composition

**DOI:** 10.1093/ofid/ofaf695.1201

**Published:** 2026-01-11

**Authors:** Giulia Orazi, Laurica A Petrella, Michelle Adamczyk, Davina Campbell, Megan Taylor, Jennifer Cadnum, Claire Kaple, Adam K Cheknis, Ashley Paulick, Stuart Johnson, Curtis Donskey, Maria Karlsson, Amy Gargis, Andrew M Skinner

**Affiliations:** Centers for Disease Control and Prevention, Atlanta, Georgia; Edward Hines Jr VA Hospital, Hines, Illinois; CDC, Atlanta, Georgia; Division of Healthcare Quality Promotion, Centers for Disease Control and Prevention, Atlanta, GA; Centers for Disease Control and Prevention, Atlanta, Georgia; Northeast Ohio VA Medical Center, Cleveland, Ohio; Case Western Reserve University, Cleveland, Ohio; Edward Hines Jr. VA Hospital, Hines, Illinois; Centers for Disease Control and Prevention, Atlanta, Georgia; Hines VA Hospital and Loyola University Medical Center, Hines, Illinois; Cleveland VA Hospital, Cleveland, Ohio; Centers for Disease Control and Prevention, Atlanta, Georgia; Centers for Disease Control & Prevnetion, Atlanta, GA; University of Utah, Salt Lake City, UT

## Abstract

**Background:**

Agar dilution is the gold standard method for performing antimicrobial susceptibility testing for *Clostridioides difficile*. Ensuring the accuracy and interlaboratory reproducibility of this method is critical, particularly in light of recent reports of isolates with reduced susceptibility to vancomycin and fidaxomicin, the two primary treatments for *C. difficile* infection. The Clinical and Laboratory Standards Institute (CLSI) recommends using Brucella agar supplemented with hemin and vitamin K_1_. We performed a multi-laboratory evaluation to investigate whether using different commercially available Brucella agars influences vancomycin minimum inhibitory concentrations (MIC) obtained by agar dilution.

**Table 1.**
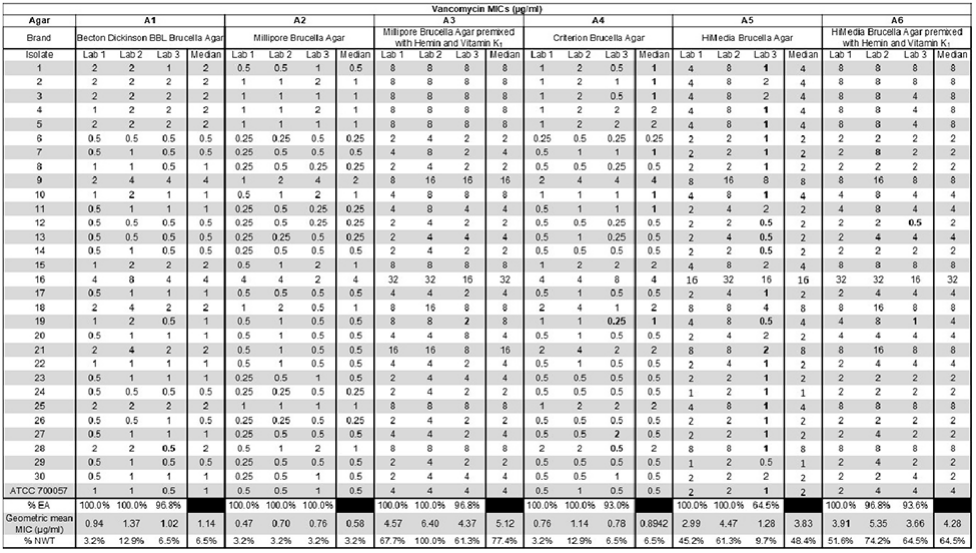
Reference agar dilution results obtained by three independent laboratories Vancomycin minimum inhibitory concentrations (MICs) of 30 clinical isolates and one QC strain were determined using six different Brucella agar formulations (A1-6): two pre-mixed with hemin and vitamin K1 (A3 and A6) and four supplemented with hemin and vitamin K1 according to CLSI recommendations (A1, A2, A4, and A5). MICs >2 log2 dilutions from the median MIC are shown in bold. MIC, minimum inhibitory concentration; EA, essential agreement (i.e., within ±1 log2 of the median MIC); NWT, non-wild type (MIC > 2 µg/ml); ATCC, American Type Culture Collection

**Methods:**

Reference agar dilution testing of 30 *C. difficile* clinical isolates and one Quality Control (QC) strain (ATCC 700057) was performed at each of three participating laboratories. Six Brucella agars from four different manufacturers were tested (A1-6): two agars preformulated with hemin and vitamin K_1_ (A3 and A6) and four supplemented by each laboratory according to CLSI recommendations (A1, A2, A4, and A5).

**Results:**

For five agars, interlaboratory reproducibility was high ( >90% essential agreement [EA] to median MIC at each lab, Table 1). Poor reproducibility was observed for A5 (Lab 3: 64.5% EA). For all labs, A3 and A6 resulted in 3-4-fold higher geometric mean MICs compared to A1, A2, and A4. In contrast, geometric mean MICs obtained with A5 varied between labs (4.19, 2.99, 1.23 µg/ml). Using the CLSI epidemiological cutoff for *C. difficile* and vancomycin, the proportion of non-wild type (NWT) isolates (i.e., MIC >2 µg/ml) ranged from 3.2-12.9% for A1, A2, and A4, whereas a higher proportion was observed for A3, A5, and A6 (9.7-100% NWT). Despite these differences, QC strain results for all labs were within the CLSI-established range.

**Conclusion:**

Our observation that agar composition may influence whether an isolate exhibits an elevated MIC to vancomycin is concerning. Because agar dilution testing results are used to identify emerging resistance and inform empirical treatment guidelines, standardization of this method is crucial. To ensure reliable results and interlaboratory comparability going forward, the methodology and QC ranges may need to be revisited.

**Disclosures:**

Andrew M. Skinner, MD, Recursion Pharmaceuticals: Advisor/Consultant

